# doubletD: detecting doublets in single-cell DNA sequencing data

**DOI:** 10.1093/bioinformatics/btab266

**Published:** 2021-07-12

**Authors:** Leah L Weber, Palash Sashittal, Mohammed El-Kebir

**Affiliations:** Department of Computer Science, University of Illinois at Urbana-Champaign, Urbama, IL 61801, USA; Department of Computer Science, University of Illinois at Urbana-Champaign, Urbama, IL 61801, USA; Department of Aerospace Engineering, University of Illinois at Urbana-Champaign, Urbana, IL 61801, USA; Department of Computer Science, University of Illinois at Urbana-Champaign, Urbama, IL 61801, USA

## Abstract

**Motivation:**

While single-cell DNA sequencing (scDNA-seq) has enabled the study of intratumor heterogeneity at an unprecedented resolution, current technologies are error-prone and often result in doublets where two or more cells are mistaken for a single cell. Not only do doublets confound downstream analyses, but the increase in doublet rate is also a major bottleneck preventing higher throughput with current single-cell technologies. Although doublet detection and removal are standard practice in scRNA-seq data analysis, options for scDNA-seq data are limited. Current methods attempt to detect doublets while also performing complex downstream analyses tasks, leading to decreased efficiency and/or performance.

**Results:**

We present doubletD, the first standalone method for detecting doublets in scDNA-seq data. Underlying our method is a simple maximum likelihood approach with a closed-form solution. We demonstrate the performance of doubletD on simulated data as well as real datasets, outperforming current methods for downstream analysis of scDNA-seq data that jointly infer doublets as well as standalone approaches for doublet detection in scRNA-seq data. Incorporating doubletD in scDNA-seq analysis pipelines will reduce complexity and lead to more accurate results.

**Availability and implementation:**

https://github.com/elkebir-group/doubletD.

**Supplementary information:**

Supplementary data are available at *Bioinformatics* online.

## 1 Introduction

The increased use of single-cell sequencing for cancer research is providing a wealth of new insights regarding intratumor heterogeneity, metastasis and the landscape of the tumor microenvironment (Gawad *et al.*, 2014; Lim *et al.*, 2020; Miles *et al.*, 2020; Morita *et al.*, 2020). In particular, the ongoing improvement in single-cell DNA sequencing (scDNA-seq) assays is rapidly advancing methods for reconstructing the evolutionary history of a tumor (El-Kebir, 2018; Jahn *et al.*, 2016; Ross and Markowetz, 2016; Roth *et al.*, 2016; Satas *et al.*, 2020; Zafar *et al.*, 2019). While scDNA-seq is more labor intensive and error-prone than traditional bulk DNA sequencing (Pellegrino *et al.*, 2018), scDNA-seq permits the observation of mutation co-occurrence patterns within a single cell, yielding both higher fidelity tumor phylogeny reconstructions and more accurate identification of a set of distinct tumor clones or genotypes.

The smaller amount of DNA material within a cell compared to RNA poses additional sequencing challenges than those faced in single-cell RNA-sequencing (scRNA-seq) (De Bourcy *et al.*, 2014). Medium to high coverage scDNA-seq technology, suitable for detecting single-nucleotide variants, suffers from elevated rates of technical errors due to whole-genome amplification that may impact downstream analyses, including allelic dropout (ADO), copying mistakes in the amplification reaction, unbalanced amplification and doublets. Specifically, when ADO occurs, one or more of the alleles may fail to be amplified during the early stages of the process and thus the allele is said to ‘drop out’ prior to sequencing. While technological advances have decreased the frequency of these errors, one remaining technical challenge is when multiple cells, or *multiplets*, are captured within a droplet and linked to a single barcode making all subsequent reads appearing as if they originated from one cell. To mitigate this effect, practitioners utilize a Poisson distribution to estimate the probability that a droplet contains a specified number of cells. The rate parameter of the Poisson distribution is then determined by a function of the cell solution concentration and droplet volume to obtain the desired probability of multiplets (Liu *et al.*, 2020). This results in the majority of droplets containing zero cells and multiplets with more than two cells are rare. However, *doublets*, which are droplets containing two cells, occur frequently and are therefore the focus of this work (Kuipers *et al.*, 2017a; Navin and Chen, 2016; Zafar *et al.*, 2018).

**Fig. 1.**
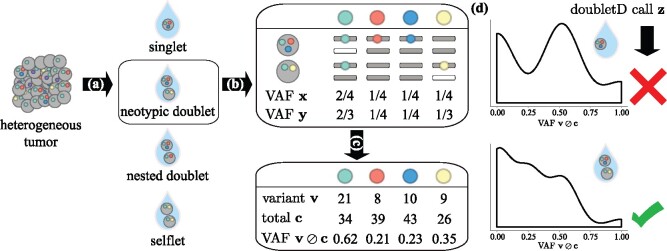
doubletD calls doublets in medium to high coverage scDNA-seq data. (**a**) The first step of most single-cell sequencing technologies involves cell capture where the goal is to encapsulate single cells into droplets, known as singlets. However, errors in this process (details in Section 1) can lead to three kind of doublets—neotypic doublets, nested doublets and selflets. (**b**) The cells in each isolated droplet *i* undergo whole-genome amplification and sequencing independently. These processes introduce errors such as ADOs and imbalance in amplification. (**c**) The resulting aligned reads are used for variant calling yielding alternate $v_{i,j}$ and total $c_{i,j}$ read counts at each locus of interest *j*. (d) doubletD uses the observed variant allele frequencies $v_{i,j}/c_{i,j}$ as the key signal, while accounting for sequencing and amplification errors to detect doublets in the sample. The symbol ⊘ denotes element-wise division

Adapting terminology from the scRNA-seq literature (Wolock *et al.*, 2019), we introduce three categories for doublets in scDNA-seq: (i) selflet, (ii) nested and (iii) neotypic (Fig. 1a). *Selflets* are comprised of cells with identical genotypes. *Nested* doublets occur when the set of mutations in one cell is a proper subset of the mutations in the other cell. A *neotypic* doublet is a doublet that is not nested or a selflet and implies the existence of a novel genotype not present in the sample. Neotypic doublets thus distort the signal of mutation co-occurrence patterns and makes it challenging to distinguish the presence of rare clones, that may be resistant to certain treatments, from a neotypic doublet (Pellegrino *et al.*, 2018). Although nested doublets and selflets will not impact the analysis of mutation co-occurrence or mutual exclusivity patterns, they may impact the estimation of clonal abundances, which are used to model both the evolutionary trajectory and the fitness landscape of a tumor (Miles *et al.*, 2020; Salehi *et al.*, 2020).

While there are downstream analysis methods, such as genotype and/or phylogeny inference methods, that account for the presence of doublets, to the best of our knowledge, there exists no standalone method for doublet detection in scDNA-seq data. There are a number of drawbacks to methods that jointly infer the doublets during any downstream analysis. First, methods like ∞SCITE (Kuipers *et al.*, 2017b), SCG (Roth *et al.*, 2016) and SiCloneFit (Zafar *et al.*, 2019) utilize Bayesian inference in the form of Markov chain Monte Carlo (MCMC) or variational inference, which scale poorly with the inclusion of doublets and size of the input (Kuipers *et al.*, 2017b; Roth *et al.*, 2016; Zafar *et al.*, 2019). Second, methods, such as ScisTree (Wu, 2020), are able to identify doublets only under the infinite sites model of evolution. Third, most methods require a binarized or discretized experiment by loci matrix input as opposed to positional variant and reference allele read counts. This results in the loss of useful information for doublet identification. Lastly, as a result of the discrete input and/or utilizing the infinite sites assumption, methods that do identify doublets are at best only able to identify neotypic doublets.

In contrast, there exist a number of standalone methods for detecting doublets in single-cell RNA-sequencing data (DePasquale *et al.*, 2019; McGinnis *et al.*, 2019; Wolock *et al.*, 2019). See Xi and Li (2020) for an excellent overview and benchmarking of scRNA-seq doublet detection methods. Doublets in single-cell RNA-sequencing (scRNA-seq) result in the observation of neotypic gene expression profiles, which impacts cell clustering and the identification of cell-state trajectories (Xi and Li, 2020). In general, these methods follow a four-step process. First, simulated doublets are created by mixing observed gene expression profiles. Second, the observed and simulated data are embedded into a latent space using dimensionality reduction. Third, machine learning methods are used to estimate the probability that a droplet is a doublet. Finally, a threshold scheme is enacted based on knowledge of the experimental doublet rate to classify experiments as either a singlet or doublet. The main variation within these methods is the choice of embedding/dimension reduction and classifier. Additionally, these methods are designed to capture neotypic doublets and struggle to identify embedded doublets, which are often located within clusters of singlets in the embedded space. While it is possible to directly apply scRNA-seq doublet detection methods on DNA variant read counts, such methods do not properly account for the distinct error profile of scDNA-seq data.

As a first step in addressing the need for a fast, standalone method for scDNA-seq doublet detection, we introduce doubletD, which performs doublet detection in medium to high coverage scDNA-seq data. Critically, doubletD does not make any assumptions about the model of evolution, the number of distinct clones or assume a threshold on the minimum clonal abundance in the sample. doubletD operates directly on variant and reference allele counts without the need to discretize the input, thus retaining a critical signal for doublet detection in the form of the variant allele frequency (VAF) (Fig. 1c). Specifically, underlying doubletD is the observation that doublets in scDNA-seq data have a characteristic VAF spectrum due to increased number of copies and/or ADO (Fig. 1d). Others have noted the presence of some of these characteristics in a *post hoc* analysis of either single-nucleotide variant (Luquette *et al.*, 2019) or copy-number aberration (CNA) calling (Zaccaria and Raphael, 2020). doubletD considers each droplet independently but borrows strength from the entire dataset while using a maximum likelihood approach in order to rapidly classify an experiment as either a doublet or singlet prior to downstream analyses. We demonstrate on both simulated and real datasets that these design choices allow doubletD to be utilized in conjunction with any downstream analysis of choice and therefore obviates the need for more complex downstream methods to individually account for the presence of doublets within their own models.

## 2 Materials and methods

### 2.1 Generative model

**Fig. 2.**
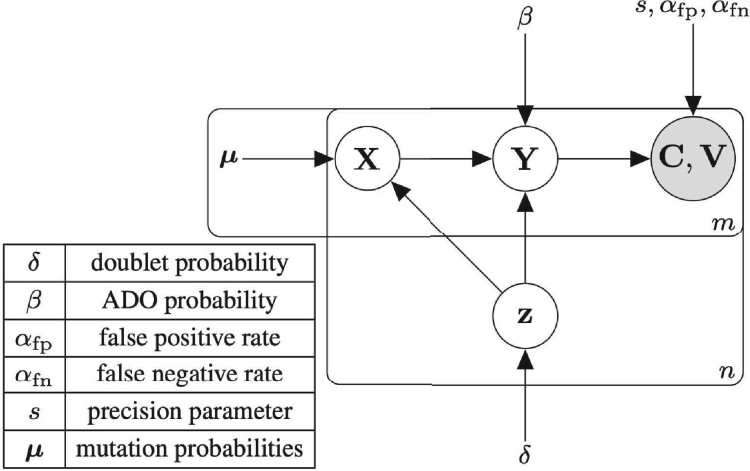
Plate diagram of doubletD ’s graphical model. Observed total and variant read counts (C,V) of *m* loci in *n* droplets are affected by doublet status z, ADO and additional errors during sequencing

Similarly to scRNA-seq, there are two main types of high-throughput cell capture strategies in scDNA-seq: microfluidics and well-based protocols, which, respectively, distribute a cell suspension into either droplets or wells (Chen *et al.*, 2019; Hwang *et al.*, 2018). Here, we use the term ‘droplet’ independent of the used technology. Consider a scDNA-seq experiment with *n* droplets and *m* mutation loci that were identified after read alignment and variant calling. Each mutation locus has two alleles: a reference allele and a variant allele. Thus, we are given $C=[c_{i,j}]\in N^{n\times m}$ total read counts and $V=[v_{i,j}]\in N^{n\times m}$ variant counts, which are independent across droplets and loci. Read counts $v_{i,j}$ and $c_{i,j}$ of mutation locus *j* in droplet *i* are affected by (i) whether droplet *i* is a doublet (Section 2.1.1), (ii) the genotype(s) at locus *j* in the droplet (Section 2.1.2), and errors during sequencing including (iii) ADO (Section 2.1.3) and (iv) amplification bias and sequencing errors (Section 2.1.4). We make these relationships explicit in a generative model for C and V (Fig. 2).

#### 2.1.1 Doublet model

In the following, we will define random variables $z\in {\left\{0,1\right\}}^{n}$, where *z_i_* indicates whether droplet *i* is a doublet (i.e. *z_i_* = 1) or a singlet (i.e. *z_i_* = 0). During the capture step, cells are released into a nozzle with a constant rate *r* and there is a fixed time-interval *t* in which a droplet is formed. The number of cells in a droplet is given by the number of cells that enter the nozzle in the time-interval during which the droplet is formed. Therefore, the prior on the doublet probability is a Poisson distribution with mean λ=rt. Moreover, only nonempty droplets will yield sequence reads. This combined with the fact that doublets are composed of two cells, we have that *z_i_* = 1, i.e. the event of droplet *i* being a doublet, equals

$$P(z_{i}=1)=\frac{\Lambda (2;\lambda )}{\sum_{k=1}^{\infty }\Lambda (k;\lambda )}=\frac{\Lambda (2;\lambda )}{1-\Lambda (0;\lambda )},$$

where Λ(k;λ) is the probability of k∈N occurrences (here cells) under a Poisson distribution with mean *λ*. In practice *rt* is very small (i.e. λ≪1), and thus the mass of the Poisson distribution Λ(k;λ) is concentrated around two outcomes k∈{1,2}. Therefore, *z_i_* can be approximately modeled by a Bernoulli distribution with probability of success δ=Λ(2;λ)/(Λ(1;λ)+Λ(2;λ)) so that

$$P(z_{i}=1)=\delta .$$

Considering independence between distinct droplets, we get

(1) $$P(z)=\prod_{i=1}^{n}\delta^{z_{i}}{\left(1-\delta \right)}^{\left(1-z_{i}\right)}.$$

#### 2.1.2 Genotype model

We make the simplifying assumption that each mutation locus has copy number 2 in a single cell—we show robustness of violations to this assumption in Section 3.1. Thus the genotype of a locus *j* in a single cell can be in one of three states: (i) wild-type (wt) where both copies have the reference allele, (ii) heterozygous (het) with one variant and one reference copy and (iii) homozygous (hom) where both copies have the variant allele. Let $\mu_{\text{wt},j},\;\mu_{\text{het},j}$ and $\mu_{\text{hom},j}$ be the mutation probabilities at locus *j* of the three types, respectively, such that $\mu_{\text{wt},j}+\mu_{\text{het},j}+\mu_{\text{hom},j}=1$. Let $x_{i,j}$ indicate the VAF at locus *j* in droplet *i*. In case *i* is a singlet, we have that $x_{i,j}\in \Sigma_{\text{singlet}}$ where $\Sigma_{\text{singlet}}=\{0,1/2,1\}$ for any locus *j*. On the other hand, if *i* is a doublet, we have that $x_{i,j}\in \Sigma_{\text{doublet}}$ where $\Sigma_{\text{doublet}}=\{0,1/4,1/2,3/4,1\}$ for any locus *j*. For a droplet *i* comprising of a single cell (*z_i_* = 0), the probability $P(x_{i,j}|z_{i}=0)$ equals

$$P(x_{i,j}|z_{i}=0)=\{\begin{array}{cc} \mu_{\text{wt},j}, & \text{if}\;x_{i,j}=0,\\ \mu_{\text{het},j}, & \text{if}\;\;x_{i,j}=1/2,\\ \mu_{\text{hom},j}, & \text{if}\;x_{i,j}=1,\\ 0, & \text{otherwise}. \end{array}$$

Following current single-cell literature (Gerstung *et al.*, 2012; Zafar *et al.*, 2016), we assume that a doublet contains two cells with independent genotypes. Therefore, we may define $P(x_{i,j}|z_{i}=1)$ using probabilities $P(x_{i,j}|z_{i}=0)$ as

$$\frac{\sum_{g,h\in S(f)}P(x_{i,j}=g|z_{i}=0)P(x_{i,j}=h|z_{i}=0)}{\sum_{g,h\in \Sigma_{\text{singlet}}\times \Sigma_{\text{singlet}}}P(x_{i,j}=g|z_{i}=0)P(x_{i,j}=h|z_{i}=0)},$$

where $S(f)=\{(g,h)\in \Sigma_{\text{singlet}}\times \Sigma_{\text{singlet}}|2g+2h=4f\}$ gives all pairs (*g*, *h*) of VAFs in $\Sigma_{\text{singlet}}$ that result in the doublet VAF *f*. For example, a doublet VAF *f* = 1/2 results from two cells with pairs (*g*, *h*) of VAFs in the set S(1/2)={(1/2,1/2),(1,0),(0,1)}.

#### 2.1.3 ADO model

We follow the work in Posada (2020) and Zafar *et al.* (2016) to model the shift in VAF due to ADOs. In this model, ADO is introduced by deciding for each cell whether a given allele is amplified or not according to a specific probability *β* known as the ADO rate. Dropout of distinct alleles is assumed to be independent and the ADO rate *β* is assumed to be constant for all cells and all loci. Although this could be easily extended to account for site-specific ADO as considered in other work (Lähnemann *et al.*, 2020); here, we opt for a global ADO rate to reduce the number of parameters. The VAF $y_{i,j}$ at locus *j* in droplet *i* after the dropout event depends on the VAF $x_{i,j}$ and doublet indicator *z_i_* (Fig. 2). Specifically, each possible pair $\left(x_{i,j},z_{i}\right)$, where $x_{i,j}\in \Sigma_{\text{singlet}}$ when *z_i_* = 0 and $x_{i,j}\in \Sigma_{\text{doublet}}$ when *z_i_* = 1, can yield varying $y_{i,j}$ with probabilities that depend on the number of alleles that are dropped during amplification. Using that each mutation locus has copy number 2 in a single cell and allowing any number of copies to drop out, we have $y_{i,j}\in \Theta_{\text{singlet}}$ where $\Theta_{\text{singlet}}=\{0,1/2,1\}$ if droplet *i* is a singlet. Conversely, if *i* is a doublet, we have $y_{i,j}\in \Theta_{\text{doublet}}$ where $\Theta_{\text{doublet}}=\{0,1/4,1/3,1/2,2/3,3/4,1\}$. Supplementary Table S1 lists all values of $P(y_{i,j}|x_{i,j},z_{i})$ for varying $\left(x_{i,j},z_{i}\right)$ and given ADO rate *β*. Supplementary Figure S1 shows an illustrative example of ADO.

#### 2.1.4 Read count model

Beyond ADO, there are two types of additional errors that affect read counts $\left(c_{i,j},v_{i,j}\right)$ and lead to an observed VAF $v_{i,j}/c_{i,j}$ that differs from the latent VAF $y_{i,j}$ after ADO: (i) copy errors, which occur early during PCR and lead to a propagation of incorrect nucleotides, and (ii) allelic imbalance, where amplification is biased toward one of the alleles (De Bourcy *et al.*, 2014). We model the resulting overdispersion with a beta-binomial as is standard in the field (Gerstung *et al.*, 2012; Lähnemann *et al.*, 2020; Zafar *et al.*, 2016). We use an uninformative prior on total read counts $c_{i,j}$ yielding

$$P(c_{i,j},v_{i,j}|y_{i,j})=P(v_{i,j}|c_{i,j},y_{i,j})P(c_{i,j})\propto P(v_{i,j}|c_{i,j},y_{i,j}).$$

While copy errors and uneven amplification errors happen simultaneously during the amplification stage, here, following Lähnemann *et al.* (2020), we employ a simpler model that assumes that the copy errors precede the allelic imbalance during amplification. We capture copy errors using a specified false positive rate $\alpha_{\text{fp}}$, which is the probability of generating an alternate allele in the copy when the template has the reference allele, and false negative rate $\alpha_{\text{fn}}$, which is the probability of generating a reference allele in the copy when the template has the alternate allele. Specifically, the probability $p_{i,j}$ of producing a copy with the alternate allele at locus *j* in experiment *i* is given by

$$p_{i,j}=y_{i,j}(1-\alpha_{\text{fn}})+(1-y_{i,j})\alpha_{\text{fp}}=\alpha_{\text{fp}}+(1-\alpha_{\text{fp}}-\alpha_{\text{fn}})y_{i,j}.$$

The number $v_{i,j}$ of variant reads resulting after amplification in the presence of allelic imbalance is modeled by the following beta-binomial distribution

$$\begin{array}{c} \pi_{i,j}∼\text{beta}(p_{i,j},s),\\ v_{i,j}|c_{i,j},\pi_{i,j}∼\text{Binom}(c_{i,j},\pi_{i,j}), \end{array}$$

where *s* is the precision parameter that quantifies allelic imbalance error. A low precision *s* signifies high unevenness in amplification.

### 2.2 Posterior probability

To determine which droplets are doublets, we are interested in the posterior probability of z for the given single-cell sequencing data (C,V), which is defined as

(2) $$P(z|C,V)=\frac{P(C,V|z)P(z)}{P(C,V)}\propto P(C,V|z)P(z).$$

In line with current methods (Zafar *et al.*, 2016, 2019), we use independence of read counts across mutation loci and droplets and obtain

$$P(C,V|z)=\prod_{i=1}^{n}\prod_{j=1}^{m}P(c_{i,j},v_{i,j}|z_{i}).$$

We now express $P(c_{i,j},v_{i,j}|z_{i})$ in terms of $P(x_{i,j}|z_{i})$ (described in Section 2.1.2), $P(y_{i,j}|x_{i,j},z_{i})$ (described in Section 2.1.3) and $P(c_{i,j},v_{i,j}|y_{i,j})$ (described in Section 2.1.4). Marginalizing over $x_{i,j}$ and $y_{i,j}$ yields

$$\begin{array}{c} P(c_{i,j},v_{i,j}|z_{i})=\sum_{x_{i,j}\in \Sigma_{i}}\sum_{y_{i,j}\in \Theta_{i}}P(c_{i,j},v_{i,j},x_{i,j},y_{i,j}|z_{i})\\ =\sum_{x_{i,j}\in \Sigma_{i}}\sum_{y_{i,j}\in \Theta_{i}}P(c_{i,j},v_{i,j}|x_{i,j},y_{i,j},z_{i})P(x_{i,j},y_{i,j}|z_{i})\\ =\sum_{x_{i,j}\in \Sigma_{i}}\sum_{y_{i,j}\in \Theta_{i}}P(c_{i,j},v_{i,j}|y_{i,j})P(y_{i,j}|x_{i,j},z_{i})P(x_{i,j}|z_{i}), \end{array}$$

where

$$\Sigma_{i}=\{\begin{array}{c} \Sigma_{\text{singlet}},\;\text{if}\;z_{i}=0,\\ \Sigma_{\text{doublet}},\;\text{otherwise}. \end{array}\;\;\text{and}\;\;\Theta_{i}=\{\begin{array}{c} \Theta_{\text{singlet}},\;\text{if}\;z_{i}=0,\\ \Theta_{\text{doublet}},\;\text{otherwise}. \end{array}$$

### 2.3 doubletD

Our goal is to find $z\in {\left\{0,1\right\}}^{n}$ such that the likelihood function [Equation (2)] is maximized. Substituting the doublet prior from Equation (1) in Equation (2) and taking log , we get

(3) $$\text{log}\;P(z|C,V)=\sum_{i=1}^{n}\sum_{j=1}^{m}\;\text{log}\;P(c_{i,j},v_{i,j}|z_{i})+\sum_{i=1}^{n}\;\text{log}\;P(z_{i})+K$$,

where *K* is the constant of proportionality. Since *z_i_* is an indicator variable (i.e. $z_{i}\in \{0,1\}$), we linearize the above equation in terms of z using

$$\text{log}\;P(c_{i,j},v_{i,j}|z_{i})=\text{log}\;P(c_{i,j},v_{i,j}|z_{i}=0)+z_{i}\Omega_{i,j},$$

where

$$\Omega_{i,j}=\text{log}\;\left(\frac{P(c_{i,j},v_{i,j}|z_{i}=1)}{P(c_{i,j},v_{i,j}|z_{i}=0)}\right)$$

and

 logP(zi)=logP(zi=0)+zilogP(zi=1)logP(zi=0)=logPzi=0+zi log δ1-δ,

where the last equality uses doublet prior model [Equation (1)]. Note that, since the read counts $\left(c_{i,j},v_{i,j}\right)$ are observed, the matrix $\Omega =[\Omega_{i,j}]\in R^{n\times m}$ is constant. Ignoring the constant of proportionality *K*, which is independent of z, and using linearization of $\text{log}\;P(c_{i,j},v_{i,j}|z_{i})$ and $\text{log}\;P(z_{i})$ in Equation (3), we get the following linear objective function:

$$J(z)=\Phi +\sum_{i=1}^{n}z_{i}\left(\sum_{j=1}^{m}\Omega_{i,j}+\text{log}\;\left(\frac{\delta }{1-\delta }\right)\right),$$

where Φ is a constant defined as follows:

$$\Phi =\sum_{i=1}^{n}\sum_{j=1}^{m}\;\text{log}\;P(c_{i,j},v_{i,j}|z_{i}=0)+\sum_{i=1}^{n}\;\text{log}\;P(z_{i}=0).$$

Since J(z) is linear, we have the following closed-form solution maximizing J(z)
 

$$z_{i}=\{\begin{array}{c} 1,\;\text{if}\;\sum_{j=1}^{m}\Omega_{i,j}+\text{log}\;\left(\frac{\delta }{1-\delta }\right)>0,\\ 0,\;\text{otherwise}. \end{array}$$

#### 2.3.1 Implementation details

Our resulting method, doubletD, identifies $z\in {\left\{0,1\right\}}^{n}$ given total and variant read counts (C,V) with maximum posterior probability P(z|C,V). To do so, doubletD requires input mutation probabilities $\mu_{\text{wt}},\mu_{\text{het}}$ and $\mu_{\text{hom}}$ at each locus *j* used in the genotype model (Section 2.1.2), and the precision parameter *s* used in the read count model (Section 2.1.4). Supplementary Appendix A.1 describes a data-driven approach to estimate these parameters. Moreover, the doublet prior probability *δ* can either be taken as input or estimated by maximizing the posterior probability. doubletD is implemented in Python 3, is open source (BSD-3-Clause license), and is available at https://github.com/elkebir-group/doubletD.

## 3 Results

We evaluated the performance of doubletD via *in-silico* experiments with known ground-truth doublets (Section 3.1) as well as two real datasets: (i) a two cell line mixture (Section 3.2) and (ii) six patients with acute lymphoblastic leukemia (Gawad *et al.*, 2014) (Section 3.3).

### 3.1 *In-silico* experiments

We aim to answer the following questions: (i) Is doubletD agnostic to the choice of scDNA-seq assay and experimental design? (ii) How robust is doubletD to the presence of CNAs? (iii) Will the removal of doublets improve downstream analyses? To this end, we simulated scDNA-seq data of 10 genotypes under an evolutionary model that incorporates CNAs and SNVs, varying the number of SNVs m∈{10,50,100}, the doublet probability δ∈{0.1,0.2,0.4}, the mean sequencing coverage c∈{10×,50×,100×} and ADO probability β∈{0.0,0.05,0.25}. Each combination of simulation parameters was replicated with five different random number generator seeds, amounting to a total of 405 experiments. In each experiment, we simulated 500 *in-silico droplet*. We benchmarked our method against SCG (Roth *et al.*, 2016), a genotyping method for scDNA-seq data whose model optionally incorporates doublet detection, which we refer to as SCG:doublet, and Scrublet (Wolock *et al.*, 2019), a standalone doublet detection method designed for scRNA-seq data. We were not able to benchmark against SiCloneFit (Zafar *et al.*, 2019) and ∞SCITE (Kuipers *et al.*, 2017b), which are tree inference methods that also incorporate doublets, due to their prohibitive runtimes when run in doublet mode. Supplementary Appendix B.1 further details the simulation design, evolutionary model and method arguments. In particular, for SCG, we performed 25 restarts unless specified otherwise, using the restart with the maximum evidence lower bound (ELBO).

#### 3.1.1 Assay and design agnosticism

**Fig. 3.**
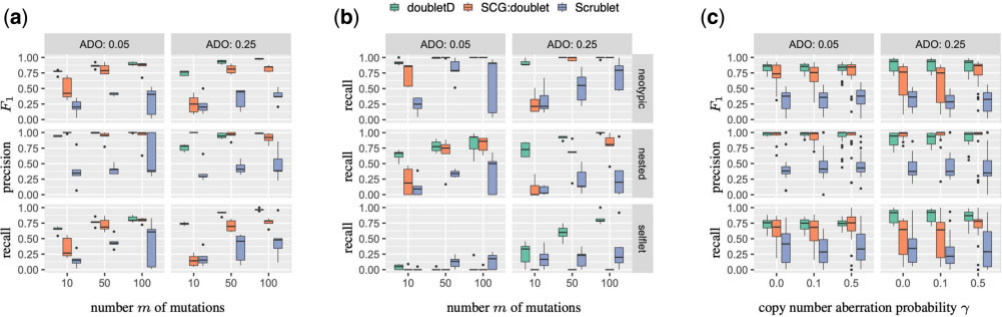
Simulations show that doubletD has high recall and precision in doublet detection, outperforming SCG and Scrublet across various experimental regimes and improving performance in downstream genotyping. (**a**) *F*_1_ score, precision and recall of doublet detection for the three competing methods (doubletD, SCG:doublet and Scrublet) in simulations with varying ADO rate *β* and number of mutations *m* in the absence of CNAs (*γ* = 0). (**b**) Recall of the three kind of doublets, i.e. neotypic, nested and selflet. (**c**) *F*_1_ score, precision and recall by method in the presence of CNAs (γ∈{0,0.1,0.5}) and varying ADO rate *β*. All results are for simulations with doublet probability δ=0.2, mean read depth c=50× and precision parameter *s* = 15

We focus on simulations with a mean coverage of c=50× and simulated doublet probability of δ=0.2. We refer to Supplementary Appendix B.1 for other simulation regimes. While all three methods show increasing *F*_1_ scores (the harmonic mean between precision and recall) with increasing number *m* of mutations, doubletD achieves the highest *F*_1_ score (median: 0.88) compared to SCG:doublet (median: 0.76) and Scrublet (median: 0.37) (Fig. 3a). Specifically, we find that Scrublet has the worst performance in terms of both recall (median: 0.35) and precision (median: 0.38), demonstrating that doublet detection methods developed for scRNA-seq data *cannot* be directly applied to scDNA-seq data. While both doubletD and SCG:doublet have equivalently high precision (SCG:doublet median: 0.99 versus doubletD median: 0.98), doubletD has superior recall (median: 0.78) among all methods (median recall of 0.67 for SCG:doublet and 0.35 for Scrublet). Strikingly, SCG:doublet performs poorly in the regime of a small number *m* = 10 of mutations, with a median recall and precision of 0.21 and 1.00, compared to 0.70 and 0.87 for doubletD, respectively. Such small number of mutations do occur in practice—e.g. the ALL data analyzed in Section 3.3.

Zooming in on doublet type in Figure 3b, we find that all methods have the highest recall for neotypic doublets (median: 1.00 for doubletD, 1.00 for SCG:doublet and 0.50 for Scrublet), and that the recall increases for both nested and neotypic doublets with increasing number of mutations and increasing ADO. Notably, doubletD has the highest recall for nested doublets (median: 0.85) compared to SCG:doublet (median: 0.57) and Scrublet (median: 0.15). As expected, doubletD and SCG:doublet are unable to detect selflets for ADO rate 0.05 while Scrublet does detect a small proportion of selflets (median: 0.05). However, when ADO rate is 0.25, doubletD has significantly higher recall (median: 0.6) as compared with SCG:doublet (median: 0) and Scrublet (median: 0.2). Note that SCG:doublet is unable to detect selflets due to VAF discretization. Further, both SCG:doublet (IQR: 0.34–0.80) and Scrublet (IQR: 0.13–0.50) show large variance in recall rates as opposed to doubletD (IQR: 0.73–0.92).

Additionally, we find that our method maintains its good performance in simulations when varying coverage and doublet probabilities (Supplementary Fig. S2). The lower bound of coverage for the *in-silico* experiments was 10×. Even at such a low coverage, doubletD maintains its good performance (median precision: 0.83 and median recall 0.78, see Supplementary Fig. S2a). It is also important to note that doubletD’s improved performance does not come at the expense of running time (Supplementary Fig. S4a, median: 14.9 s versus 11,000.0 s for SCG:doublet and 4.1 s for Scrublet). Finally, doubletD is robust to the choice of user-specified parameters such as the precision *s* (Supplementary Appendix B.1.4, Supplementary Figs S5–S8). In summary, we find that doubletD is robust to many variations in experimental assays and design, outperforming SCG:doublet and Scrublet.

#### 3.1.2 Robustness with respect to CNAs

In order to evaluate the robustness of doubletD to the presence of CNAs, we generated simulations with varying probability of CNAs γ∈{0,0.1,0.5}, where *γ *= 0 represents simulations with no CNAs. More specifically, for each locus that undergoes a CNA (with probability *γ*), we introduced a loss with probability ℓ∈{0.1,0.5} and a gain otherwise. We ran SCG:doublet with five restarts due to increased runtimes compared to the copy-neutral simulations.

Although doubletD does not explicitly account for CNAs, Fig. 3c shows that doubletD is robust to varying CNA probability *γ*, outperforming SCG:doublet and Scrublet in most regimes. Specifically, doubletD yields the highest recall (median: 0.79) with good precision (median: 0.98) resulting in the highest *F*_1_ score (median: 0.87) compared to SCG:doublet (median: 0.80) and Scrublet (0.36). While SCG:doublet has the same precision as doubletD (median: 0.98), this comes at the cost of lower recall (median: 0.73) compared to doubletD (median: 0.79).

The robustness of doubletD can be explained by the observation that losses (deletions) introduced by CNAs behave similarly to ADOs, which is a key signal used by doubletD to detect doublets. We demonstrate the vulnerability of doubletD to copy number gains on simulations with highest possible CNA probability *γ *= 1 and lowest possible loss probability ℓ=0 (Supplementary Fig. S3). Note that this kind of extreme presentation of CNAs is not observed in practice and that copy number losses including loss of heterozygosity events are common in cancer (El-Kebir, 2018; McPherson *et al.*, 2016; Satas *et al.*, 2020).

In summary, we find that doubletD is robust to the presence of CNAs and outperforms both SCG:doublet and Scrublet in doublet detection.

#### 3.1.3 Improving downstream genotype calling

SCG is a genotyping method for scDNA-seq data of tumors that includes doublet detection. It has two modes: in *singlet mode* (SCG:singlet) all droplets are considered singlets, whereas in *doublet mode* (SCG:doublet) genotypes and doublets in the sample are jointly inferred. Here, we assess whether the sequential use of doubletD followed by SCG:singlet (doubletD + SCG:singlet) performs better than SCG:singlet and SCG:doublet. In each of these settings, SCG is run with 25 restarts.

**Fig. 4.**
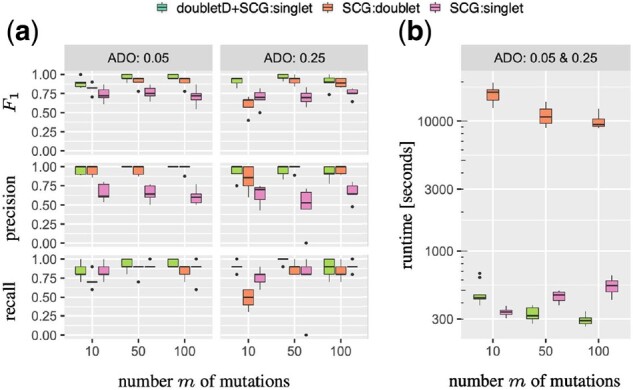
Simulations show that removal of doublets using doubletD improves downstream genotype calling with reduced runtime. (**a**) *F*_1_ score, precision and recall of genotypes for doubletD + SCG:singlet, SCG:doublet and SCG:singlet for varying number of mutation *m* and ADO rate *β* and without CNAs (*γ* = 0). (**b**) Running time for genotype calling using doubletD + SCG:singlet, SCG:doublet and SCG:singlet for simulations with varying number of mutations *m* without CNAs (*γ* = 0). All results are for simulations with doublet probability δ=0.2, mean read depth c=50× and precision parameter *s* = 15

Recall that each of our simulated instances contain 10 genotypes. To assess the performance of the three methods, we compute recall, precision and *F*_1_ score with respect to these ground-truth genotypes, considering a genotype as correctly inferred (i.e. a true positive) if it precisely matches a ground-truth genotype. Thus, if a method infers the exact set of 10 ground-truth genotypes, its recall, precision and *F*_1_ score will be 1. We find that doubletD + SCG:singlet has the highest *F*_1_ score (median: 0.95) compared to SCG:singlet (median: 0.73) and SCG:doublet (median 0.89) across all experimental regimes (Fig. 4a). SCG:singlet has good genotype recall (median: 0.9) but reduced precision (median: 0.64) since it misidentifies doublets as cells with distinct genotypes. SCG:doublet, on the other hand, has better precision (median: 1.0) but filters out rare genotypes misidentified as doublets resulting in reduced recall (median: 0.80). doubletD + SCG:singlet yields the highest recall (median: 0.90) and precision (median: 1.0). In general, SCG:singlet calls more genotypes (median: 14) while SCG:doublet calls fewer genotypes (median: 8.5) compared to the ground truth of 10 genotypes (Supplementary Fig. S9). On the other hand, doubletD + SCG:singlet is closer to ground truth with a median of 9.5 distinct genotypes. Furthermore, Fig. 4b shows that doubletD + SCG:singlet takes orders of magnitude less time compared to SCG:doublet. While SCG:singlet takes the least time to run, it also yields the lowest *F*_1_ score (Fig. 4a).

In summary, we find that the use of doubletD improves genotype calling of SCG while incurring runtimes comparable to SCG in singlet mode. This suggests that doublet removal using doubletD is a useful preprocessing step for downstream analyses of scDNA-seq data of tumors.

### 3.2 Mixture of two cell lines

We validated doubletD on a dataset of n=1569 droplets comprised of a 50–50% mix of KG-1 and Raji cell lines (with *m* = 26 loci) captured by Mission Bio’s Tapestri platform and sequenced by Illumina NextSeq (https://portal.missionbio.com/datasets/KG-1-Raji-50-50-Myeloid). Supplementary Appendix B.2 details the data preparation, including the exclusion of 23 cells that had a genotype distinct from the two cell lines. KG-1 had 12 heterozygous (het), 7 wt and 7 homozygous loci, while Raji had 11 heterozygous, 7 wt and 8 homozygous loci (Supplementary Fig. S10). The mean sequencing coverage *c* was 110×. Following the procedure outlined in Supplementary Appendix A.1, we fit beta-binomial precision *s* = 10.5, $\alpha_{\text{fp}}=0.015,\;\alpha_{\text{fn}}=0.0073$ and locus-specific mutation probabilities μ to the observed variant V and total read counts C. We used the experimental ADO rate (β=0.06) previously estimated by Morita *et al.* (2020) on a large patient cohort using Mission Bio’s Tapestri platform.

There are two unique characteristics of this dataset that permit identification of neotypic doublets for orthogonal validation: (i) the droplets are easily clustered into two clones by the cell line of origin (Supplementary Fig. S10) and (ii) the droplets are comprised of distinct cell lines with distinct evolutionary histories. These characteristics are uncommon in regular datasets where the number of clones and associated genotypes is unknown *a priori* and droplets originate from a single tumor whose clones have a shared evolutionary history. As such, we conclude that doublets will be either neotypic (one cell from each cell line), or selflets (two cells from one cell line).

**Fig. 5.**
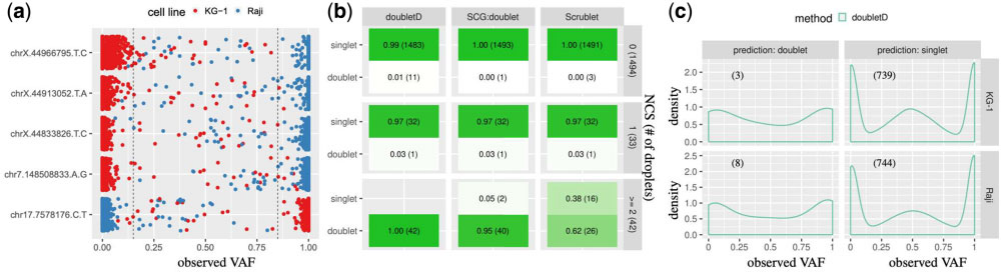
doubletD successfully recalls all 42 orthogonally validated high confidence neotypic doublets and identifies 11 putative selflets in a two cell line mixture dataset. (**a**) The VAF for each droplet at each of the five validation loci. Droplets are assigned a NCS, which is the number of validation loci whose VAF was in the range [0.15,0.85] (dotted lines). (**b**) The resulting proportion (total) of droplet calls by method (doubletD, SCG:doublet and Scrublet) by prediction (singlet, doublet) and NCS. (**c**) The aggregated observed VAF distribution by doubletD prediction and cell line for droplets with NCS=0. The number of droplets in the aggregate are shown in the parentheses

Using the property that the two cell lines have independent origins and relaxing Mission Bio’s standard filtering criteria, we identified an additional set of five *validation loci* with distinct wt/homozygous states among the two cell lines, i.e. each validation locus has state wt (hom) in one cell line and hom (wt) in the other (Fig. 5a). Recall that a singlet *i* will have an observed VAF $v_{i,j}/c_{i,j}$ of approximately 1 if locus *j* is homozygous and VAF 0 if locus *j* is wt. As such, any droplets with observed VAF not close to either 0 or 1 (Fig. 5a) indicate that the droplet may be a neotypic doublet comprised of a cell from each cell line. We therefore assign a *neotypic doublet confidence score* (NCS) to each droplet, counting the number of validation loci with VAF between 0.15 and 0.85. This approach yielded 1,494 droplets with NCS=0, 33 droplets with NCS=1 and 42 droplets with NCS≥2. Note that the NCS is specifically designed to express confidence that a doublet is neotypic but does not capture selflets. Supplementary Figure S10 shows a comparison of the observed VAF of droplets categorized by cell line droplets with a NCS≥2.

We ran doubletD, SCG:doublet (with five restarts) and Scrublet. Since we did not know the true doublet probability *δ*, we used the maximum likelihood criterion to establish the estimate the doublet probability for doubletD as δ=0.05 (Supplementary Fig. S11a). However, we provided SCG and Scrublet with the doublet probability δ=0.09 as estimated by Mission Bio in similar cell line experiments (Mission Bio, 2019). For each method and NCS, we calculated the proportion of predicted singlets and doublets (Fig. 5b). doubletD identified the most droplets as doublets (54), followed by SCG:doublet (42) and Scrublet (30). doubletD predicted 100% of doublets with NCS≥2 whereas SCG:doublet identifies 95.2% of these droplets with similarly high NCS. Scrublet is the worst performing, identifying only 61.9% of such droplets (Fig. 5b). In terms of running time, SCG:doublet took 16,259.7 s, doubletD took 24.1 s and Scrublet took 2.4 s.

All three methods designated the same droplet at NCS=1 as a doublet. This suggests that for the remaining 32 droplets at NCS=1 the observed VAF in [0.15,0.85] at one of these five validation loci is likely attributable to amplification and sequencing error. The one doublet identified by all methods does appear to be neotypic as evidenced by an observed VAF of 0.39 for the validation locus on chromosome 17, which is far from the cut off criterion of 0.15 and is hard to explain by other errors. Furthermore, the VAF distribution across the 26 inference loci for this droplet has a peak at 0.25 and is strikingly different from the distribution of the other Raji droplets with NCS equal to 1 (Supplementary Fig. S11b). Lastly, doubletD identifies 11 (proportion: 0.007) putative selflets at NCS=0, 3 of which are KG-1 and 8 are Raji. SCG calls 1, which was also called by doubletD, and Scrublet calls 3 such droplets with only one called by doubletD. Corroborating this, we note a visual difference in the aggregated VAF distribution across the inference 26 loci between doubletD predicted singlets and doublets with NCS=0 (Fig. 5c). A Venn diagram of the droplets with different NCS score that were predicted as doublets by the three competing methods is shown in Supplementary Fig. S12.

In summary, doubletD is able to recall all orthogonally validated high confidence neotypic doublets (with NCS ≥2) as well as successfully distinguish the VAF signal of neotypic doublets from sequencing-related error. In addition, we suspect that doubletD is able to recall a small number of selflets even in the presence of low ADO rates (β=0.05).

### 3.3 Phylogeny inference of an acute lymphoblastic leukemia patient

As discussed in Section 1, while nested doublets and selflets do not yield new genotypes, neotypic doublets can be mistaken as an additional clone with a unique genotype (Navin and Chen, 2016). In the extreme case of a phylogeny with only two branches, neotypic doublets that correspond to the two leaves of this tree will include all mutations. Consequently, phylogeny inference under the infinite sites assumption will yield a linear phylogeny. Here, we investigate the impact of doublets on phylogeny inference for a patient (Patient 1) in an acute lymphoblastic leukemia cohort previously suspected to contain doublet droplets (Gawad *et al.*, 2014)—we refer to Supplementary Table S2 for doubletD results of the other patients.

**Fig. 6.**
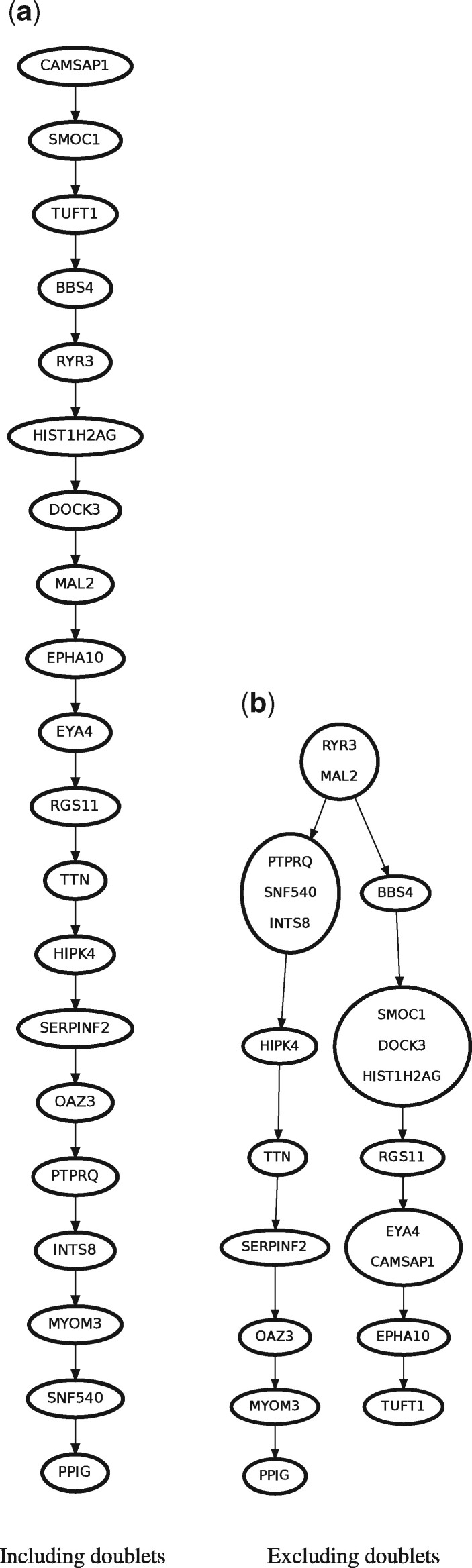
Doublets lead to incorrect phylogeny inference in acute lymphoblastic leukemia patient 1. (**a**) PhISCS-B returns a linear phylogeny with mean log likelihood of −2806.49/243=−11.55 if the 50 doublets detected by doubletD are retained. (**b**) PhISCS-B returns a branching phylogeny with higher mean likelihood of −1157.39/193=−6.00

Gawad *et al.* (2014) sequenced 243 droplets and identified 20 mutations for Patient 1. We analyzed this patient using PhISCS-B (Malikic *et al.*, 2019b), which is a phylogeny inference method that seeks to identify a tree constrained by the infinite sites assumption. Since it does not account for doublets, PhISCS-B requires doublets be removed in a preprocessing step. While SCG:doublet was unable to identify any doublets, doubletD identified 50 doublets for this patient. Supplementary Figure S13 corroborates these doublets, showing distinct VAF distributions between singlets and doublets for an orthogonal set of holdout loci. We ran PhISCS-B in single-cell data mode on the complete set of droplets (including doublets) as well as the set of droplets without doublets (details in Supplementary Appendix B.3). Figure 6 shows that doublet removal in this patient results in a branching phylogeny with a higher mean likelihood (−1157.39/193=−6.00) compared to a linear phylogeny (−2806.49/243=−11.55) on the complete set of droplets. Furthermore, the branching pattern observed in the inferred phylogeny after doublet removal is in agreement with several other trees published for Patient 1, with identical grouping of the mutations across the two branches (Gawad *et al.*, 2014; Kuipers *et al.*, 2017b; Malikic *et al.*, 2019a).

Thus, phylogeny inference is an additional example of a downstream analysis where the inclusion of doublets may yield incorrect conclusions.

## 4 Discussion

In this work, we introduced doubletD, the first standalone method for detecting doublets in scDNA-seq data with medium to high coverage (≥5×) suitable for single-nucleotide variants. Our method operates directly on variant and total read counts of mutation loci. Underlying our method is the observation that doublets in scDNA-seq data have a characteristic VAF distribution. An additional signal that we exploit is the shift in VAFs due to ADO. This unique approach enables doubletD to capitalize on a major downside of single-cell sequencing in order to identify selflets and nested doublets, that are notoriously hard to detect by current methods. doubletD utilizes a probabilistic model that specifically accounts for allelic imbalance and dropout during whole-genome amplification in scDNA-seq as well as sequencing errors. We introduced a closed-form solution for the inference problem. We demonstrated that our method outperforms current methods for downstream analysis of scDNA-seq data that jointly infer doublets and genotypes (Roth *et al.*, 2016) as well as standalone approaches for doublet detection in scRNA-seq data (Wolock *et al.*, 2019). Moreover, we showed that removing doublets using doubletD improves the accuracy and efficiency of downstream analyses such as genotype calling and phylogeny inference.

There are several opportunities for future work. First, while this paper focused on cancer, doubletD can be applied to normal samples as well using heterozygous germline SNPs. Moreover, the same characteristic signal used by our method to detect doublets can be used to detect cells that have undergone whole-genome duplication or are in S-phase with actively replicating DNA. Second, our approach can be extended to support low (0.1−0.5×) to ultra-low (<0.05×) coverage scDNA-seq samples, suitable for CNAs, by pooling heterozygous germline SNPs located within haplotype blocks. Third, our current formulation assumes that normal cells are diploid. As noted in our simulations, performance slightly decreased in the presence of CNAs. We plan to extend our probabilistic model to account for copy number. Finally, we envision that doubletD will improve downstream analysis of current and future methods, making doublet detection and removal a standard practice in scDNA-seq analysis.

## Funding

M.E-K. was supported by the National Science Foundation under award numbers CCF 1850502 and CCF 2046488.

*Conflict of Interest*: none declared.

## Data availability statement

The data underlying this article are available at https://github.com/elkebir-group/doubletD_data.

## Supplementary Material

btab266_Supplementary_Data
