# Supplementary material for: doubletD: detecting doublets in single-cell DNA sequencing data
Source: Bioinformatics. 2021 Jul 12;37(Suppl 1):i214–21. doi: 10.1093/bioinformatics/btab266 (PMC8275324; doi:10.1093/bioinformatics/btab266)
Supplement: btab266_Supplementary_Data [file btab266_supplementary_data.pdf]

Supplementary Material – doubletD: Detecting doublets in single-cell DNA sequencing data

Leah L. Weber [1,*], Palash Sashittal [2,*] and Mohammed El-Kebir [1,†]

[1]Department of Computer Science, University of Illinois at Urbana-Champaign, Urbama, IL 61801, USA. and
[2]Department of Aerospace Engineering, University of Illinois at Urbana-Champaign, Urbana, IL 61801, USA

Contents

A Supplementary Methods **2**
A.1 Parameter estimation . . . 3

B Supplementary Results **4**
B.1 Simulations . . . 4
B.1.1 Simulation setup . . . 5
B.1.2 Evolutionary model . . . 5
B.1.3 Previous methods . . . 5
B.1.4 Sensitivity to input parameters . . . 5
B.2 Two cell line mixture . . . 6
B.3 Acute lymphoblastic leukemia tumors . . . 6
B.3.1 Preprocessing . . . 6
B.3.2 Doublet detection . . . 6
B.3.3 Phylogeny inference with PhISCS-B . . . 7

*these authors contributed equally.
†To whom correspondence should be addressed.

A Supplementary Methods

- Table S1 shows the value of $P(y_{i,j} \mid x_{i,j}, z_i)$ for all possible combinations of post-ADO VAF $y_{i,j}$, pre-ADO VAF $x_{i,j}$ and doublet status z_i.
- Fig. S1 gives a schematic representation of possible outcomes of ADO for two example droplets.

$x_{i,j}$	z_i \ $y_{i,j}$	0	1/4	1/3	1/2	2/3	3/4	1	NaN
0	0	$1-\beta^2$	0	0	0	0	0	0	β^2
1/2	0	$\beta(1-\beta)$	0	0	$(1-\beta)^2$	0	0	$\beta(1-\beta)$	β^2
1	0	0	0	0	0	0	0	$1-\beta^2$	β^2
0	1	$1-\beta^4$	0	0	0	0	0	0	β^4
1/4	1	$\beta(1-\beta)^3+$ $3\beta^2(1-\beta)^2+$ $3\beta^3(1-\beta)$	$(1-\beta)^4$	$3\beta(1-\beta)^3$	$3\beta^2(1-\beta)^2$	0	0	$\beta^3(1-\beta)$	β^4
1/2	1	$\beta^2(1-\beta)^2+$ $2\beta^3(1-\beta)$	0	$2\beta(1-\beta)^3$	$(1-\beta)^4+$ $4\beta^2(1-\beta)^2$	$2\beta(1-\beta)^3$	0	$\beta^2(1-\beta)^2+$ $2\beta^3(1-\beta)$	β^4
3/4	1	$\beta^3(1-\beta)$	0	0	$3\beta^2(1-\beta)^2$	$3\beta(1-\beta)^3$	$(1-\beta)^4$	$\beta(1-\beta)^3+$ $3\beta^2(1-\beta)^2+$ $3\beta^3(1-\beta)$	β^4
1	1	0	0	0	0	0	0	$1-\beta^4$	β^4

Table S1: This table shows the value of $P(y_{i,j} \mid x_{i,j}, z_i)$, i.e. the probability of having VAF $y_{i,j}$ at locus j in droplet i after allelic dropout (ADO) given pre-ADO VAF $x_{i,j}$ and doublet status z_i. The last column 'NaN' represents the case when all the alleles are dropped and, as a result, no reads span locus j in droplet i. The values in each row sum to 1. Schematic representations of allelic dropout in a singlet and a doublet droplet are shown in Fig. S1.

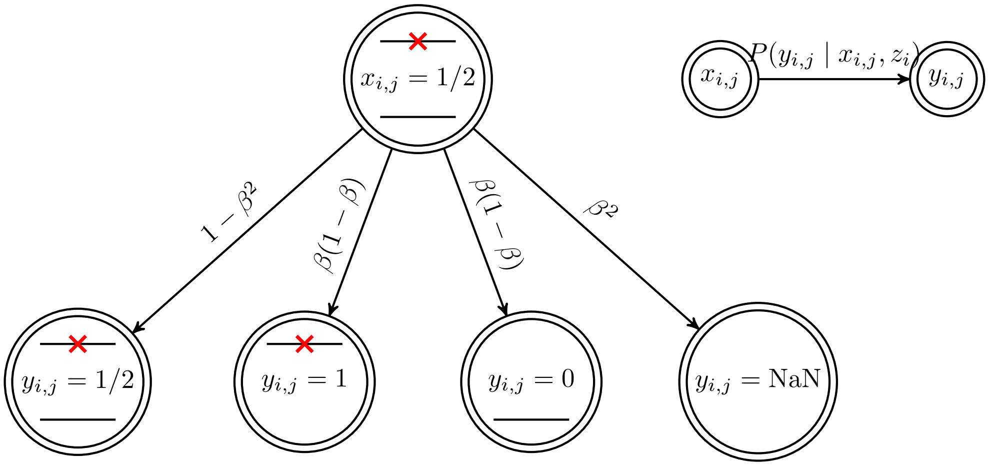

(a) $z_i = 0$

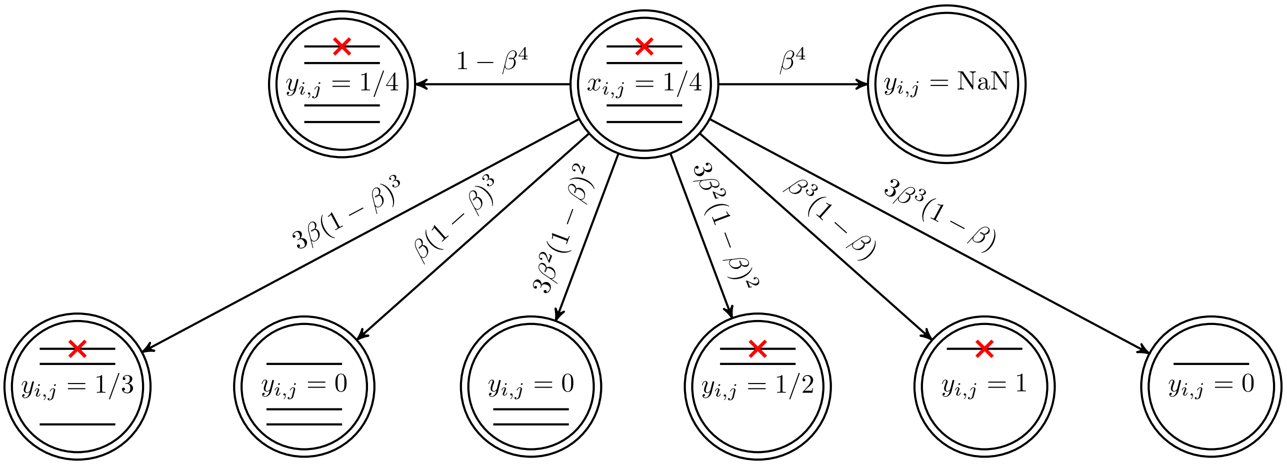

(b) $z_i = 1$

Figure S1: Schematic representation of allelic dropout in a droplet with (a) single cell with a heterozygous mutation and (b) doublet composed of two cells, one with a heterozygous mutation and other with two wild-type alleles. The circle represents the droplet and the black lines inside the droplet indicate copies of the genome. We consider all cells to be diploid and therefore each cell contributes two copies of the genome. The red cross represents a variant allele. The expression on the arrows depict the probabilities $P(y_{i,j} \mid x_{i,j}, z_i)$. The values of $P(y_{i,j} \mid x_{i,j}, z_i)$ are shown in Table S1.

A.1 Parameter estimation

doubletD requires the user to input mutation probabilities $\mu_{\text{wt}}, \mu_{\text{het}}$ and μ_{hom} at each locus j used in the genotype model (Section 2.1.2), and the precision parameter s used in the read count model (Section 2.1.4). In this section we describe a data-driven approach to estimate these parameters.

Due to the evolutionary pressures on the cells in the sample, the rate of mutations can change significantly across loci. We therefore use the data to get the mutation probabilities $\mu_{\text{wt},j}, \mu_{\text{het},j}$ and $\mu_{\text{hom},j}$ for each locus j which serve as the input parameters for the genotype model (Section 2.1.2). The observed VAFs $v_{i,j}/c_{i,j}$ for each droplet i at locus j are mapped to the closest value in Σ_{singlet}. For $x \in \Sigma_{\text{singlet}}$, let $d_j(x)$ be the number of cells in

which the VAF at site j was mapped to x. We estimate the mutation rates as follows,

$$\mu_{\mathrm{wt},j} = \frac{d_j(0)}{d_j(0) + d_j(1/2) + d_j(1)},$$
$$\mu_{\mathrm{het},j} = \frac{d_j(1/2)}{d_j(0) + d_j(1/2) + d_j(1)},$$
$$\mu_{\mathrm{hom},j} = \frac{d_j(1)}{d_j(0) + d_j(1/2) + d_j(1)}.$$

Fig. S8a shows that this method gives reliable estimates of the mutation probabilities in simulations.

To estimate the precision parameter s for the beta-binomial distribution, the observed VAFs $v_{i,j}/c_{i,j}$ for each droplet i at locus j are mapped to a value in $\Sigma_{\mathrm{singlet}}$. For each $x \in \Sigma_{\mathrm{singlet}}$, let ω_x be the the set of observed VAFs mapped to x. We first fit shape parameters $\hat{\alpha}_x$, $\hat{\beta}_x$ for each $x \in \Sigma_{\mathrm{singlet}}$ set ω_x, utilizing method of moments estimation (Owen, 2008) and obtain observed precision $\hat{s}_x = \hat{\alpha}_x + \hat{\beta}_x$. Since our method utilizes a global precision parameter for all droplets and loci, we set the precision parameter to the median of the set $\{\hat{s}_x \mid x \in \Sigma_{\mathrm{singlet}}\}$. The estimation of this parameter can be supplemented from non-variant loci or SNP positions in addition to the observed VAFs. Fig. S8b shows that we recover reliable estimates of the precision parameter s in simulations. See Lodato *et al.* (2015) for an alternative estimation procedure and MDA specific shape parameters that scale linearly with sequencing coverage.

B Supplementary Results

B.1 Simulations

- Fig. S2 shows that doubletD maintains its good performance in simulations with varying coverage and doublet probabilities.
- Fig. S3 demonstrates the vulnerability of doubletD to copy number gains on simulations with highest possible copy number aberration probability $\gamma = 1$ and lowest possible loss probability $\ell = 0$.
- Fig. S5 shows that doubletD achieves similar precision and recall values using the maximum likelihood estimate of doublet probability δ as using the ground truth doublet rate.
- Fig. S6 shows that the maximum likelihood estimate of the doublet probability δ using doubletD is close to the ground truth doublet probabiilty in simulations.
- Fig. S7 shows that doubletD is robust to the choice of the input precision parameter s.
- Fig. S8 shows that doubletD accurately estimates the mutation probabilities $\boldsymbol{\mu}$ and the precision parameter s from read-count data.
- Fig. S4 shows that doubletD is orders of magnitude faster than SCG in simulations both with and without copy number aberrations and for varying experimental regimes.
- Fig. S9 shows that combining doubletD with SCG in singlet mode recovers closest to the ground truth number of simulated genotypes versus SCG doublet mode and SCG in singlet mode without doublet detection.
- Fig. S10 is a heatmap showing the observed variant allele frequency (VAF) of cell line droplets categorized by cell line or droplets with a neotypic doublet confidence score (NCS ≥ 2)
- Fig. S11 shows the posterior likelihood function for varying doublet probability δ and the observed VAF distributions for doubletD predicted doublets and singlets for Raji droplets with neotypic doublet confidence score NCS $= 0$.
- Fig. S12 shows a Venn diagram of the droplets with different NCS scores.
- Fig. S13 shows the aggregated observed variant allele frequency distribution for the ALL patients on the hold-out loci and the loci used for the doublet inference.

B.1.1 Simulation setup

We generate variant $\mathbf{V}$ and total read counts $\mathbf{C}$ for 500 *in silico* droplets as follows. First, we evolve 10 genotypes under an evolutionary model that incorporates CNAs and SNVs (detailed below) and use a symmetric Dirichlet distribution to obtain clonal abundances (concentration parameter $\alpha = 2$). The minimum allowable clonal abundance for any genotype was 0.02, which we enforce using rejection sampling. We vary the number of SNVs $m \in \{10, 50, 100\}$. Next, we decided for each droplet whether it is a doublet with probability $\delta \in \{0.1, 0.2, 0.4\}$. Depending on the outcome, we randomly sample one or two cells from the 10 genotypes to comprise the droplet. We also vary the mean sequencing coverage $c \in \{10, 50, 100\}$ and ADO probability $\beta \in \{0.0, 0.05, 0.25\}$. To draw total reads $\mathbf{C}$, we use a negative binomial distribution parameterized by the mean sequencing coverage c and a dispersion of 5. The copy error rate was fixed at $\alpha_{\text{fp}} = \alpha_{\text{fn}} = 0.001$ for all experiments in accordance with (De Bourcy *et al.*, 2014). Finally, we set the beta-binomial precision to $s = 15$ — a simulation regime that matches the Tapestri platform by Mission Bio (Section 3.2 as well as MDA-based single-cell DNA sequencing (Section 3.3). Using the drawn total read counts $\mathbf{C}$ and the above parameters, we draw variant read counts $\mathbf{V}$. Each combination of simulation parameters was replicated with five different random number generator seeds, amounting to a total of 405 experiments.

B.1.2 Evolutionary model

We now describe the model that we used to simulate the evolutionary history of $k = 10$ genotypes comprised of both CNAs and SNVs. Using Prüfer sequences (Prüfer, 1918), we begin by drawing a labeled tree T comprised of 10 nodes uniformly at random. For each of the m SNVs, we decide whether that mutation will undergo copy-neutral loss of heterozygosity (CN-LOH) with a probability of 0.1 (as described above). Then, we uniformly assign each mutation to a node of T to generate mutation clusters when $m > k$. For each non CN-LOH mutation, we decide with probability γ if that mutation will undergo a CNA event. These define a set of CNA events. For the set of CNA events, we then decide if that event is a loss with probability ℓ or a gain otherwise. For any gain events, we determine the number of copies gained by drawing a number uniformly between 1 and the max number of copies (3 for base simulations and 5 for our extreme CNA scenario). Next, we randomly assign CNA events to nodes of the tree T. We then generate the set of k genotypes by evolving SNV events (het or hom) and CNV events down the tree. We start with a pair (ω_i, ρ_i) representing the number of variant alleles and reference alleles of each SNV locus $i \in [m]$. At each node, we update this genotype in accordance with the events encountered on the path from the root to the node. When applying CNA events, if the current number of mutated alleles is non-zero, we select either the mutated or reference allele to undergo the CNA event. Otherwise, it is applied to the reference allele. If an SNV and CNA are introduced at the same time, we randomly determine which event to apply first.

B.1.3 Previous methods

We benchmarked our method against scrublet (Wolock *et al.*, 2019) and SCG (Roth *et al.*, 2016). We provided scrublet with our simulated variant read counts and doublet rate as input. For SCG, we discretized the input by applying the binomial exact test with 0.001 and 0.999 as the null rates for input discrete states wild type=0 and cn-LOH=2 respectively with p-values of 10^{-6} required to reject the null hypothesis in favor of the state heterozygous=1 in accordance with (Roth *et al.*, 2016). Total read depths of 0 were assigned to a missing state=3. For the symmetric Dirichlet prior, we set $\kappa = 2$ and set the doublet rate prior in accordance with the simulated doublet rate δ. All remaining parameters and inputs were left as the default values.

B.1.4 Sensitivity to input parameters

Our method takes several parameters as input (Fig. 2). The ADO probability β and the sequencing error rates $\alpha_{\text{fp}}, \alpha_{\text{fn}}$ are sequencing platform specific and typically known a priori. While the doublet probability δ is also typically known beforehand, we find that maximum likelihood is good criterion for estimating this parameter in case it is unknown. Specifically, varying the doublet probability in $\{0.01, 0.1, 0.2, 0.4, 0.7, 0.9\}$ and selecting value with maximum likelihood, we achieve similar precision and recall values as using the ground truth doublet rate (Fig. S5). Further, the likelihood function is maximized at or close to the simulated doublet rate indicating that it can be used as a reliable criteria for estimating the true doublet rate (Fig S6).

doubletD estimates the beta-binomial precision parameter s and mutation probabilities μ from input data. We find that procedure outlined in Section A.1 for estimation the beta-binomial precision parameter s and mutation probabilities μ from input data results in only minor error with a deviation of 0.08, -0.11, 0.02, and 6.0 for μ_{wt}, μ_{het} and μ_{hom} (Fig. S8a) and s (Fig. S8a), respectively. We then fixed the inference doublet rate at the simulated rate

and varied the beta precision by inputting half and twice the simulated beta precision into doubletD (Fig. S7). We found that halving or doubling in inference beta precision parameter with respect to the simulated parameter had no significant impact when the simulated precision was high (1000). When the simulated precision was low (15), we noted that overestimation by utilizing double the simulated precision parameter did result in a small decrease in the median precision of 0.86 to 0.81 (Fig. S7). Conversely, utilizing half the simulated rate resulted in improved precision without a reduction in recall. Thus, in the presence of uncertainty of this parameter, preference should be given to underestimation or lower values. In conclusion, we found that doubletD is resilient to variations in the user-inputted doublet rate parameter, especially in the range of typical experimental doublet rates (0.1-0.4). As the inference doublet rate increases beyond this range, precision is reduced since the threshold for calling an experiment a doublet is significantly lowered. The likelihood function calculated as a function of the predicted experimental labels is maximized at or close to the simulated doublet rate (Fig S5).

B.2 Two cell line mixture

We followed the procedure outlined in the vignette accompanying this dataset[1]. The one notable exception is that we used relaxed filtering criteria to identify additional loci for orthogonal doublet validation. The filtering criteria were as follows.

```
gt.filter=TRUE, gt.gqc = 30, \
gt.dpc = 10, gt.afc = 20, \
gt.mv = 50, gt.mc = 50, \
gt.mm = 0.5, gt.mask = TRUE
```

We extracted variant and total read counts from the AD and DP layers of the loom file for 1592 droplets. To identify a subset of 26 high-quality inference loci among the total number of 133 loci, we excluded loci that had a copy number greater than 2 (using the `compute_ploidy` function in the Tapestri R package).

Upon performing a preliminary dimensionality reduction (t-sne) and hierarchical clustering on the 'zygosity' (binning of VAFs into homozygous, heterozygous or wild type) of all 133 loci, we noted the existence of a third cluster composed of 23 droplets with a distinct genotype and mutually exclusive mutations from the other two cell lines. We excluded these droplets from our analyses. Further, we identified a set of 5 validation loci distinct from the 23 inference loci that were homozygous (hom) in one cell line cluster but wild type (wt) in the other. We use these loci to establish the ground truth assignment of droplets to the two cell lines, Raji and KG-1, and to compute the NCS score for each droplet.

B.3 Acute lymphoblastic leukemia tumors

B.3.1 Preprocessing

We utilized scDNA-seq data in the form of FASTQ files from the Sequence Read Archive database (accession no. SRP044380). After adaptor trimming (Trimmomatic), read alignment (bwa) to reference genome hg19 and PCR duplicate removal (Picard), we performed a pileup of the variant positions called by Gawad *et al.* (2014) to obtain the variant $\mathbf{V}$ and total read counts $\mathbf{C}$.

B.3.2 Doublet detection

To mitigate the impact of CNAs on doublet detection, we only included loci that were heterozygous and had a median VAF $\in [0.45, 0.55]$. We ran doubletD directly on the total and variant read count data obtained from the pileup utilizing the ADO rates reported by Gawad *et al.* (2014). Since no information was published on the expected doublet rate, we performed a grid search to obtain the maximum likelihood estimate of the prior doublet probability δ. Fig. S13 shows that the identified doublets have distinct VAF distributions compared to the remaining singlet droplets, both on the set of inference loci (that met the heterozygosity filtering criterion) as well as an orthogonal set of holdout loci (that did not meet the filtering criterion). Table S2 shows the statistics and results generated by applying doubletD on data from all the patients in the dataset. Section 3.3 shows detailed analysis of Patient 1.

[1] `https://support.missionbio.com/hc/en-us/articles/360045899834-Installation-instructions-for-tapestriR`

patient	n	m'	m	mean coverage	ADO β	doubletD
1	243	20	14	14.3×	0.20	50 (0.21)
2	256	16	9	15.6×	0.18	22 (0.09)
3	266	48	31	9.1×	0.25	86 (0.32)
4	276	78	50	7.1×	0.24	92 (0.33)
5	225	105	59	7.0×	0.25	55 (0.24)
6	224	10	7	16.2×	0.18	46 (0.21)

Table S2: **Statistics and doublet detection results of an acute lymphoblastic leukemia cohort of six patients.** From left to right, the table shows for each patient the number n of droplets, the number m' of loci identified by (Gawad *et al.*, 2014), the number m of loci that meet our filtering criteria, the mean coverage of the samples, the ADO rate reported by Gawad *et al.* (2014) and the number (and fraction) of doublets identified by doubletD.

B.3.3 Phylogeny inference with PhISCS-B

PhISCS-B operates on a discretized input matrix that codes for the presence ('1') or absence ('0') of a mutation in a droplet as well as missing data ('?'). To discretize the input matrix, we used the binomial exact test to determine mutation status for each corresponding entry in the total and variant read count matrices **C** and **V** with a null error rate of 0.001 and a p-value of 10^{-6}. We provided PhISCS-B with the ADO rate $\beta = 0.2$ reported by Gawad *et al.* (2014) and a false positive rate of 0.001 that is typical for multiple displacement amplification (MDA) whole-genome amplification (Fu *et al.*, 2015). We imposed a maximum time limit of 3600 s.

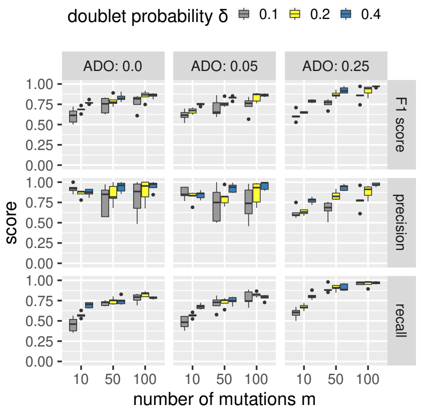

(a) $c = 10\times$

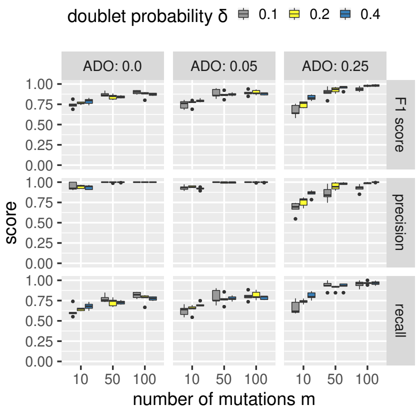

(b) $c = 50\times$

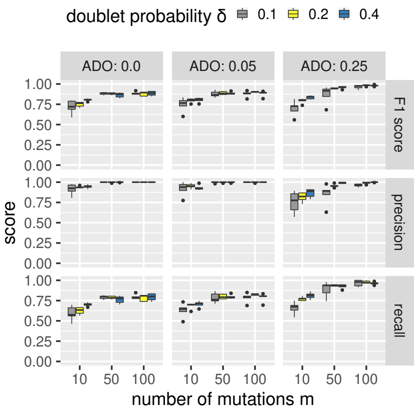

(c) $c = 100\times$

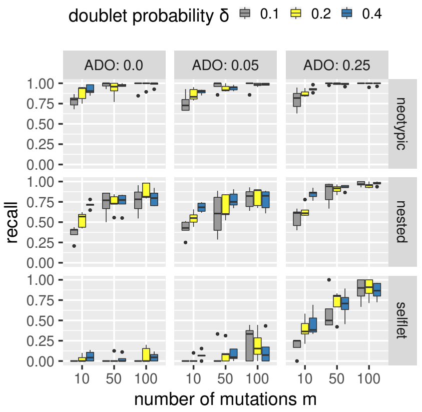

(d) $c = 10\times$

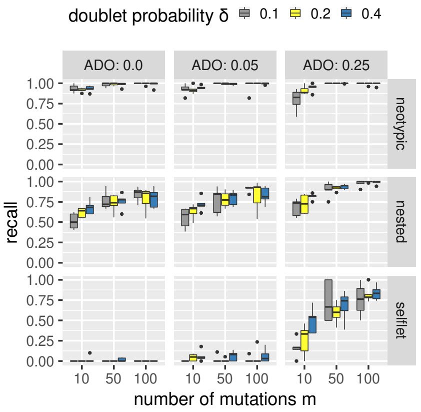

(e) $c = 50\times$

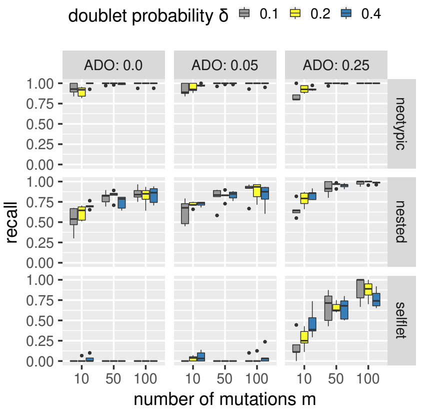

(f) $c = 100\times$

Figure S2: F_1 score, recall and precision of doublet detection using DOUBLETD for varying mean read depths c, ADO rates β and doublet probabilities δ. All simulations are run without copy number aberrations $\gamma = 0$ and precision parameter $s = 15$.

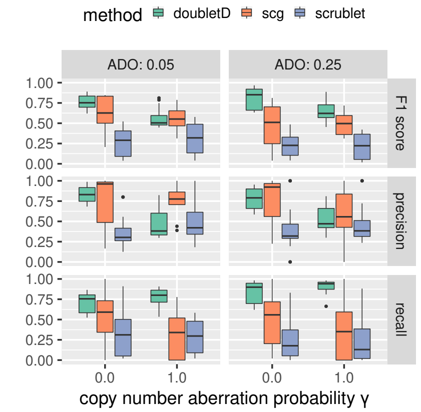

(a)

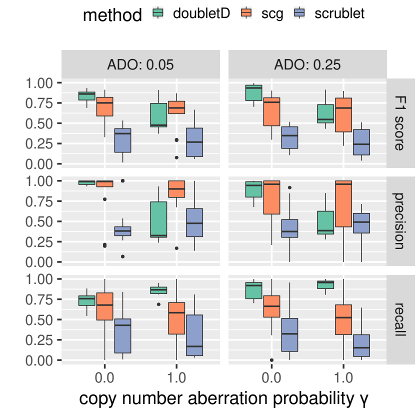

(b)

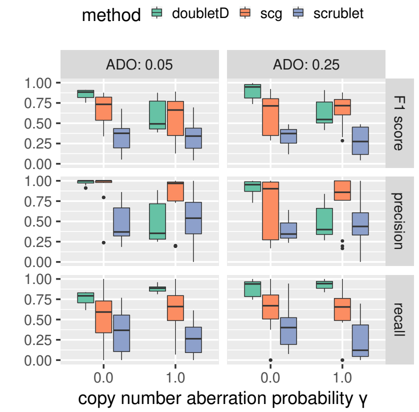

(c)

Figure S3: F_1 score, recall and precision of doublet detection using DOUBLETD on simulations without copy number losses and only gains. Results are shown for copy number aberrations probability $\gamma \in \{0, 1\}$ and ADO rates $\beta \in \{0.05, 0.25\}$. All simulations are run with doublet probability $\delta = 0.2$, mean read depth $c = 50\times$, number of mutations $m \in \{10, 50, 100\}$ and precision parameter $s = 15$.

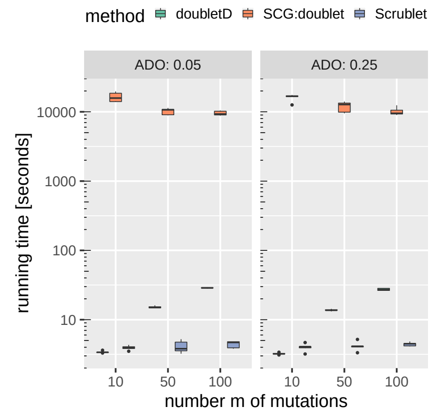

Figure S4: Running time for doublet detection using DOUBLETD, SCG:doublet and SCRUBLET for simulations without CNAs ($\gamma = 0$) with varying number of mutations m. All simulations have doublet probability $\delta = 0.2$, mean read depth $c = 50\times$ and precision parameter $s = 15$.

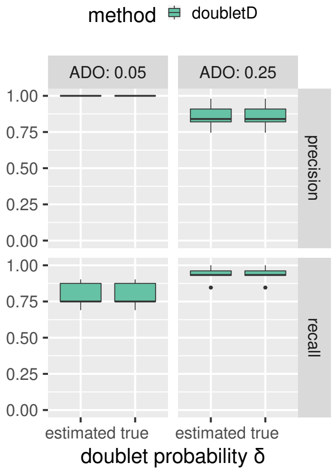

(a) $c = 10\times$

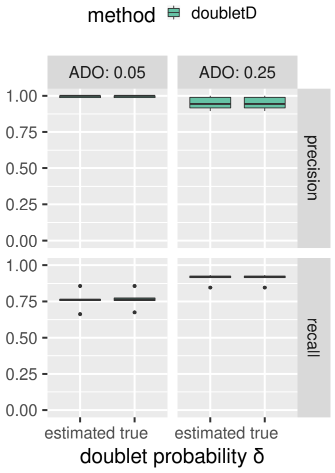

(b) $c = 50\times$

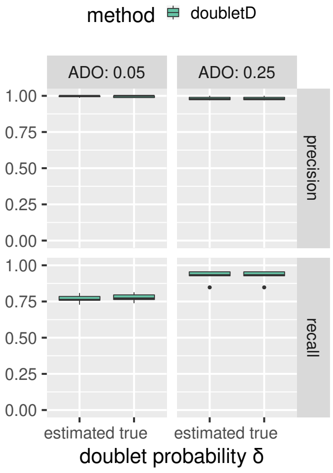

(c) $c = 100\times$

Figure S5: Precision and recall for doublet detection using DOUBLETD with maximum likelihood estimate of the doublet probability δ and the true doublet probability used in the simulations for varying ADO rates β and mean read depth c. Results are shown for simulations with doublet probability $\delta = 0.2$, number of mutations $m \in \{10, 50, 100\}$ and precision parameter $s = 15$.

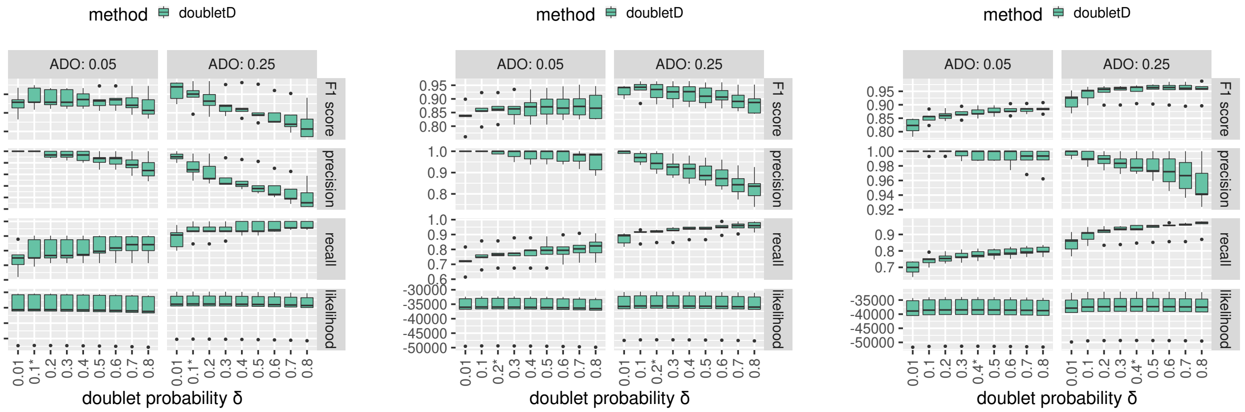

(a) (b) (c)

Figure S6: F_1 score, precision, recall and posterior likelihood of doublet detection using DOUBLETD with varying input doublet probability δ. The simulations are run with doublet probability $\delta = \{0.1, 0.2, 0.4\}$, number of mutations $m = 50$, coverage $50\times$ and precision parameter $s = 15$. Copy number aberration probability γ was set to 0.

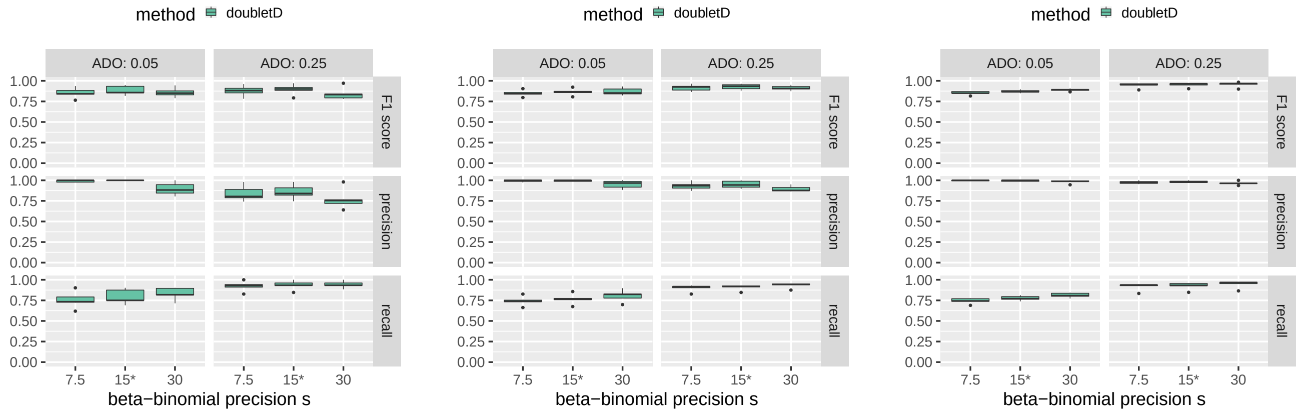

(a) (b) (c)

Figure S7: F_1 score, precision, recall and posterior likelihood of doublet detection using DOUBLETD with varying input precision parameter s. The simulations are run with doublet probability $\delta = 0.2$, number of mutations $m \in \{10, 50, 100\}$ and precision parameter $s = 15$. Copy number aberration probability γ was set to 0.

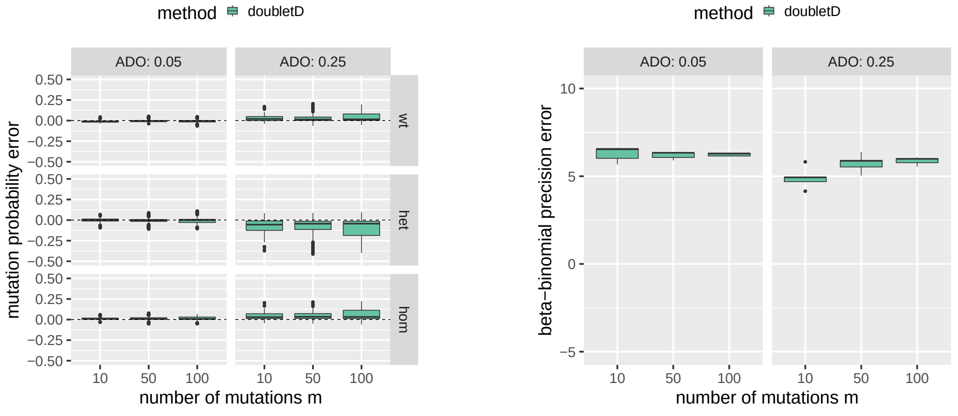

(a) (b)

Figure S8: Error in the estimation of (a) the mutation probabilities $\boldsymbol{\mu} = \{\boldsymbol{\mu}_{\mathbf{wt}}, \boldsymbol{\mu}_{\mathbf{het}}, \boldsymbol{\mu}_{\mathbf{hom}}\}$ and (b) the precision parameter s from simulated data with varying number of mutations m and ADO rates β. All simulations are run with doublet probability $\delta = 0.2$, copy number aberration probability $\gamma = 0$ and precision parameter $s = 15$.

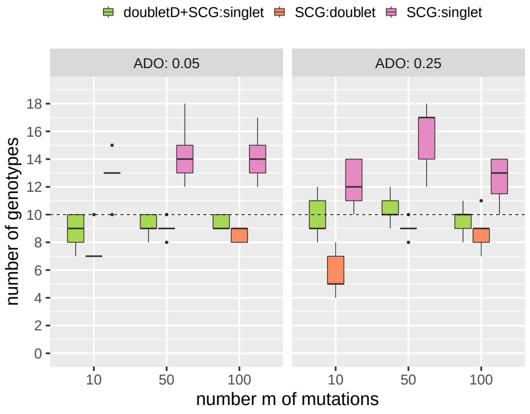

Figure S9: Number of genotypes called by doubletD+SCG:singlet, SCG:doublet and SCG:singlet on simulations with varying number of muations m and ADO rates β. All simulations have doublet probability $\delta = 0.2$, mean read depth $c = 50\times$, precision parameter $s = 15$ and copy number aberration $\gamma = 0$.

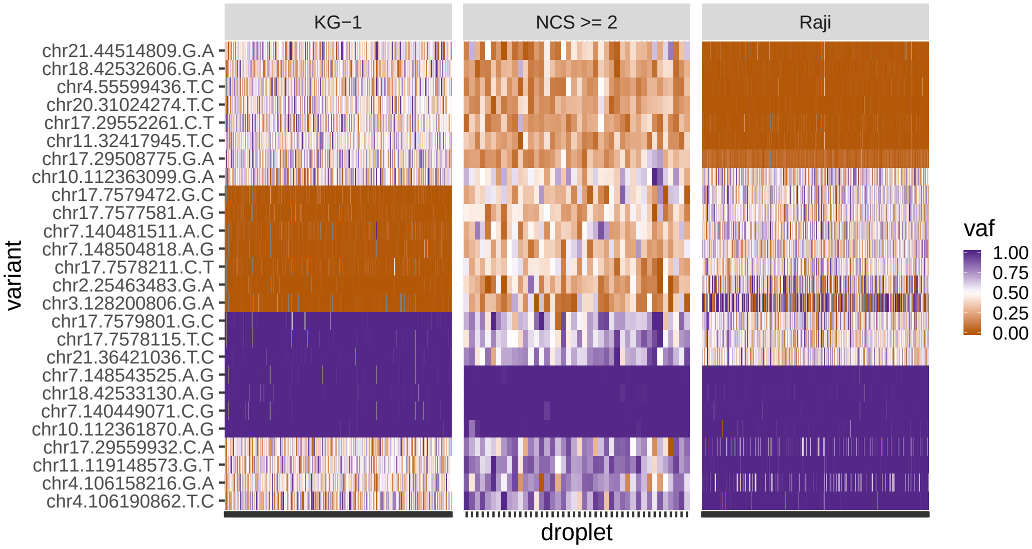

Figure S10: Heatmap showing the observed variant allele frequency (VAF) of cell line droplets categorized by cell line or droplets with a neotypic doublet confidence score (NCS ≥ 2).

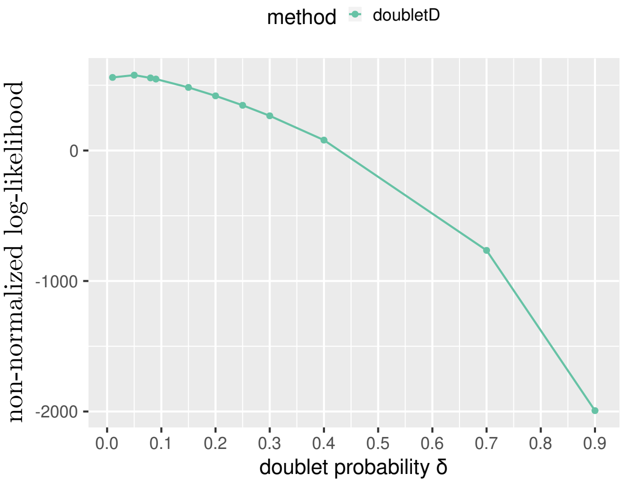

(a)

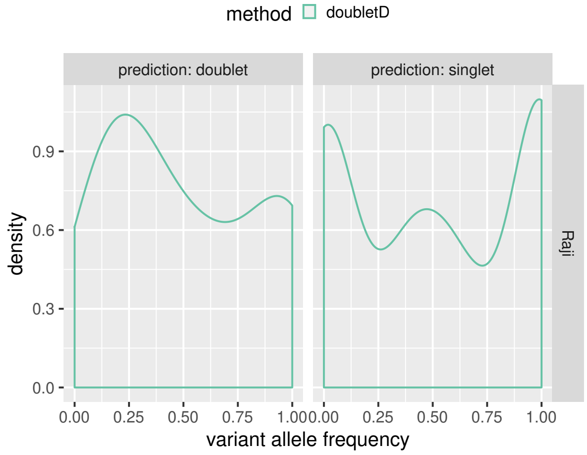

(b)

Figure S11: DOUBLETD results on cell line data. (a) The posterior likelihood (non-normalized) as a function of input doublet probability δ. Due to non-normalization, the log-likelihood is shifted by a constant explaining the positive values observed in the plot. (b) The observed VAF distributions for doubletD predicted doublets (1) and singlet droplets (8) for Raji droplets with neotypic doublet confidence score NCS $= 1$.

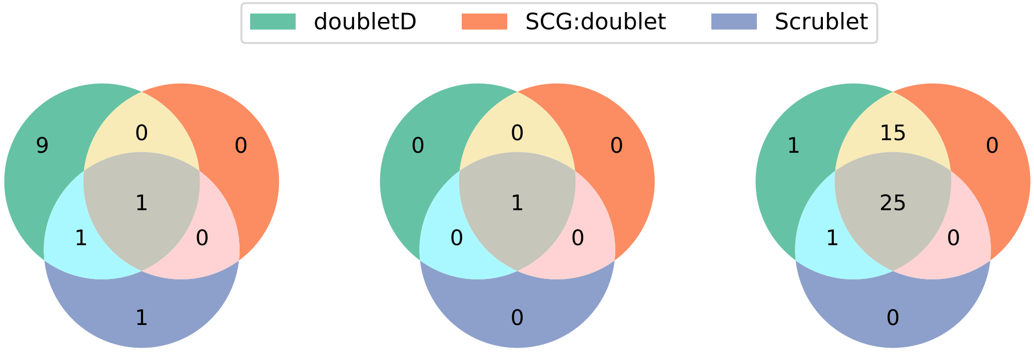

(a) NCS = 0 (b) NCS = 1 (c) NCS $\geq$ 2

Figure S12: Venn diagram of the droplets with NCS score of (a) 0 (b) 1 and (c) $\geq$ 2 that were predicted as doublets by the three methods, doubletD, SCG:doublet and Scrublet.

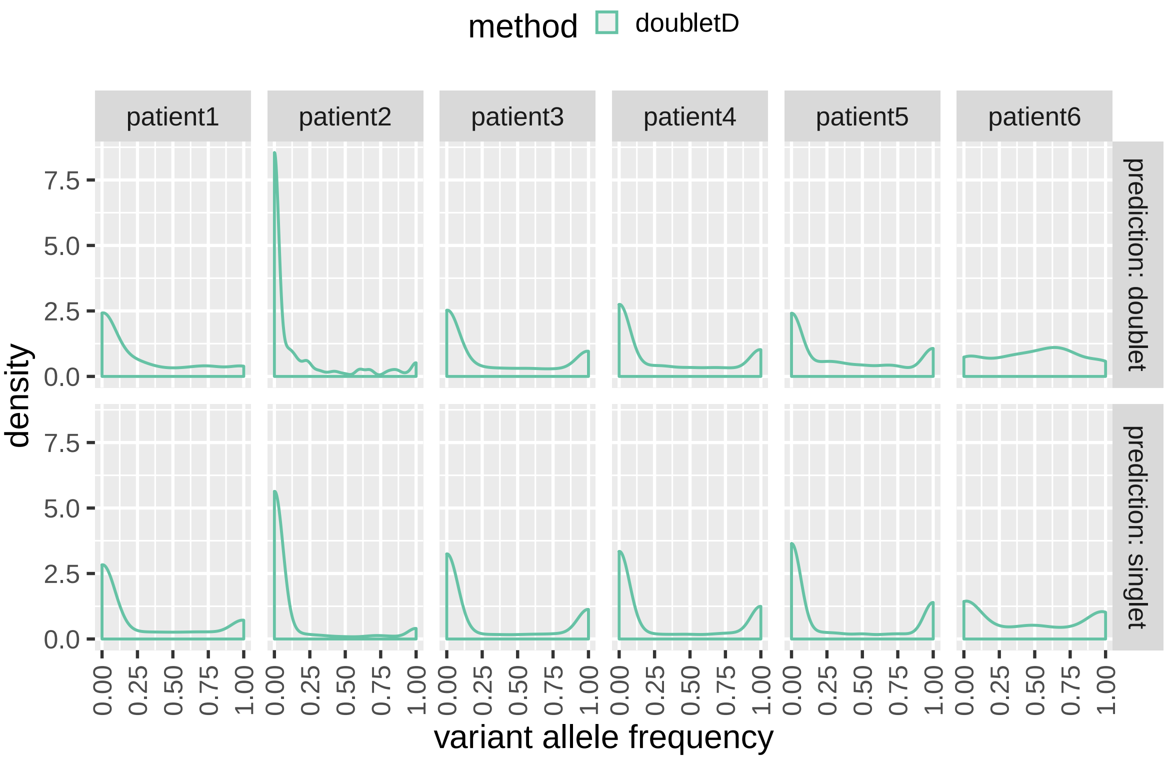

(a)

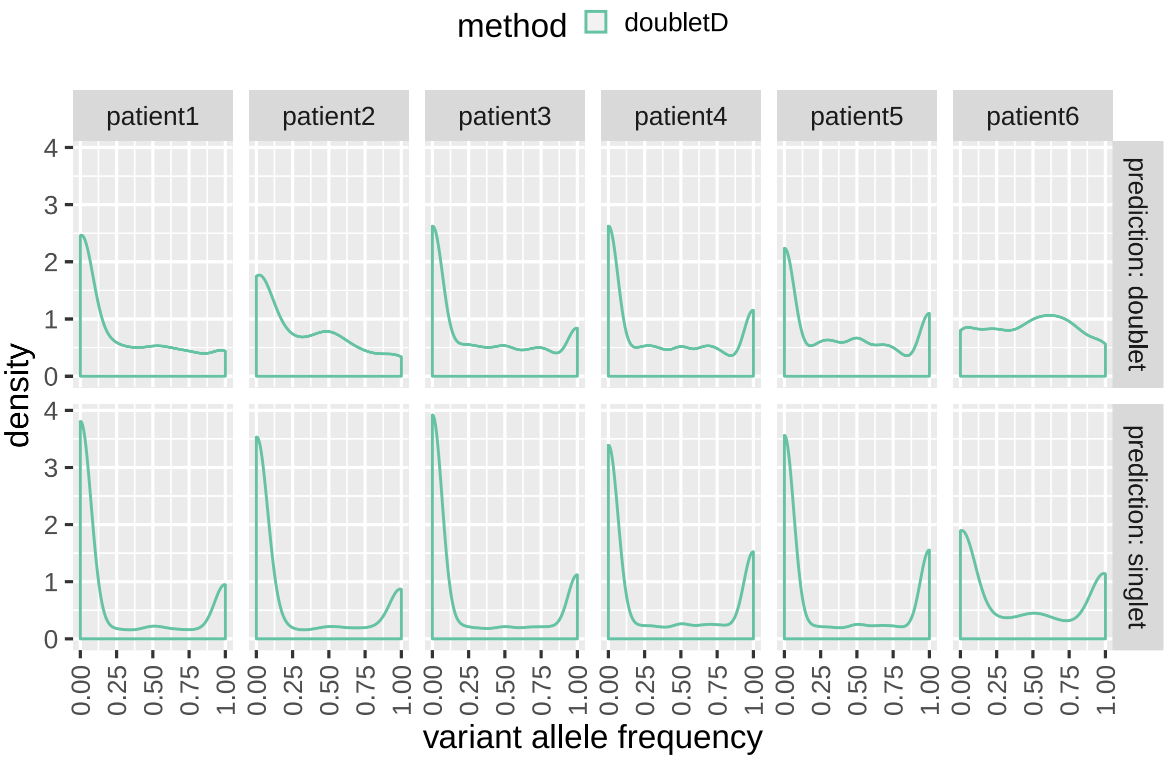

(b)

Figure S13: Aggregated observed variant allele frequency distribution by patient and DOUBLETD prediction for (a) across holdout loci (b) across inference loci.

References

De Bourcy, C. F. *et al.* (2014). A quantitative comparison of single-cell whole genome amplification methods. *PLOS One*, **9**(8), e105585.

Fu, Y. *et al.* (2015). Uniform and accurate single-cell sequencing based on emulsion whole-genome amplification. *Proceedings of the National Academy of Sciences*, **112**(38), 11923–11928.

Gawad, C. *et al.* (2014). Dissecting the clonal origins of childhood acute lymphoblastic leukemia by single-cell genomics. *Proceedings of the National Academy of Sciences*, **111**(50), 17947–17952.

Lodato, M. A. *et al.* (2015). Somatic mutation in single human neurons tracks developmental and transcriptional history. *Science*, **350**(6256), 94–98.

Owen, C. E. B. (2008). Parameter estimation for the beta distribution.

Prüfer, H. (1918). Neuer beweis eines satzes uber permutationen. *Arch Math Phys*, **27**, 742–4.

Roth, A. *et al.* (2016). Clonal genotype and population structure inference from single-cell tumor sequencing. *Nature methods*, **13**(7), 573–576.

Wolock, S. L. *et al.* (2019). Scrublet: computational identification of cell doublets in single-cell transcriptomic data. *Cell systems*, **8**(4), 281–291.
